# The relationship between paternal breastfeeding support self-efficacy and maternal breastfeeding behavior at 6 weeks postpartum: the mediating role of breastfeeding knowledge

**DOI:** 10.3389/fpsyg.2026.1915683

**Published:** 2026-10-21

**Authors:** Minghui Li, Yingping Wang, Cuiling Xie, Jingyi Li, Xiaoqian Chen, Yuqing Pan, Fang Chen, Xiumin Jiang

**Affiliations:** 1 Nursing Department, Shenzhen Bao’an District Songgang People’s Hospital, Shenzhen, Guangdong, China; 2 Nursing Department, Fujian Provincial Maternity and Children's Hospital, Fuzhou, Fujian, China

**Keywords:** breastfeeding behavior, breastfeeding knowledge, breastfeeding support, fathers, mediation analysis, self-efficacy

## Abstract

Breastfeeding is universally recognized as the optimal method of infant feeding, and fathers, as mothers’ intimate partners, serve a critical supportive function throughout the breastfeeding process; however, the specific pathways through which paternal breastfeeding support self-efficacy is associated with early postpartum breastfeeding behavior remain unclear. This cross-sectional study, conducted between February and December 2024, recruited 454 mother–father dyads at 6 weeks postpartum from a tertiary maternal and child health hospital in Fujian Province, China. Data were collected using the Paternal Breastfeeding Support Self-Efficacy Scale, the Maternal Breastfeeding Behavior Scale for the Puerperium, and the Breastfeeding Knowledge Questionnaire, and were analyzed using Pearson correlation, multiple linear regression, and structural equation modeling with bootstrap mediation testing in the lavaan package in R 4.3.1. Paternal breastfeeding support self-efficacy (PBSSE) was positively correlated with maternal breastfeeding knowledge and with maternal breastfeeding behavior at 6 weeks postpartum, and maternal breastfeeding knowledge was positively correlated with breastfeeding behavior (all *p* < 0.01). Path analysis indicated that PBSSE was indirectly associated with breastfeeding behavior at 6 weeks postpartum through maternal breastfeeding knowledge (indirect effect *β* = 0.131, 95% CI: 0.030–0.234), with the mediating effect accounting for 24.14% of the total effect; the direct effect remained significant (*β* = 0.412, *p* < 0.001), indicating partial mediation. These findings suggest that, in clinical practice, attending to fathers’ self-efficacy and strengthening breastfeeding knowledge education for mothers may help optimize breastfeeding behavior during the early postpartum period. Because the design was cross-sectional, the observed mediation should be interpreted as a statistical association rather than a causal mechanism.

## 1 Introduction

Breastfeeding is universally recognized as the optimal method of infant feeding (The Lancet, 2017). Extensive research has confirmed that breast milk provides a suitable combination of nutrients for infants, promoting gastrointestinal maturation, establishing early immune defense, reducing the risk of respiratory and gastrointestinal infections, and exerting long-term positive effects on cognitive development (Castanys-Muñoz et al., 2016; Le Doare et al., 2018). Breastfeeding is also associated with maternal health benefits, including lower risks of postpartum complications, cardiovascular disease, and breast cancer (Del Ciampo and Del Ciampo, 2018; Zachou et al., 2019). The Lancet breastfeeding series has further highlighted breastfeeding as a major public health opportunity, estimating that improved breastfeeding rates could prevent substantial child and maternal mortality worldwide and reduce public health costs (The Lancet, 2017; Victora et al., 2016). The World Health Organization recommends that infants should be exclusively breastfed as soon as possible after birth for at least 6 months, with continued breastfeeding alongside complementary foods until 2 years of age or beyond (World Health Organization, 2022). Empirical studies further highlight that early initiation, breastfeeding competency, and determinants of exclusive breastfeeding are key issues for improving breastfeeding outcomes (Alrasheedi, 2023; Shi et al., 2021; Wu, Y. L. et al., 2021). Previous studies have demonstrated that the first 6 weeks postpartum represent a critical transitional period for the successful establishment and maintenance of breastfeeding (Brown et al., 2014). During this period, women are undergoing postpartum physical and psychological recovery while assuming the maternal role (Tsai and Wang, 2019; World Health Organization, 2022). Mothers also need to acquire breastfeeding knowledge and skills, respond to breastfeeding difficulties, and gradually form stable feeding behavior patterns (Feligreras-Alcalá et al., 2021; Nakamura et al., 2018). Yet they frequently encounter physiological barriers such as insufficient milk supply, nipple pain, and infant latch difficulties, as well as psychological challenges including lack of confidence, fatigue, and anxiety (Henshaw et al., 2018; Mangrio et al., 2018; Zhang et al., 2018). These early barriers may influence breastfeeding decisions during the current postpartum period and subsequent pregnancies (Xiao et al., 2020). Consequently, support from the family, particularly from the father, is especially crucial during this period (Zhou et al., 2024).

In recent years, the supportive role of fathers in breastfeeding has received increasing scholarly attention. Paternal support has been identified as an important component of breastfeeding social support systems and is associated with improved breastfeeding outcomes and duration (Abbass-Dick et al., 2019; Zhou et al., 2024). Male-partner-focused interventions can affect breastfeeding initiation, exclusivity, and continuation (Mitchell-Box and Braun, 2013). Dennis et al. (2018) conceptualized paternal breastfeeding support self-efficacy (PBSSE)—fathers’ confidence in assisting mothers with breastfeeding—based on Bandura’s (1977) self-efficacy theory. According to this framework, individuals with higher self-efficacy are more inclined to engage in information-seeking and supportive behaviors; fathers with greater PBSSE may accordingly be more likely to acquire breastfeeding knowledge and share it with their partners. The Knowledge–Attitude–Practice (KAP) theory posits that knowledge is the foundation for behavioral change and that insufficient understanding may hinder the adoption of desired health behaviors (Launiala, 2009). Consistently, higher maternal breastfeeding knowledge has been associated with more favorable feeding practices (Blyth et al., 2002; Li et al., 2023; Wang et al., 2025; Wu, S. V. et al., 2021; Wu, W. et al., 2021). Integrating these two theoretical perspectives yields a coherent pathway: fathers with higher PBSSE may be more inclined to seek and share breastfeeding knowledge, and this enhanced maternal knowledge may in turn be associated with more favorable breastfeeding behaviors—a pathway that remains untested in previous research.

Despite growing interest in paternal involvement, existing studies have predominantly examined PBSSE and breastfeeding behavior as separate constructs, with paternal self-efficacy treated as a peripheral variable rather than a core predictor. Although Zeng et al. (2024) examined the direct association between PBSSE and exclusive breastfeeding, the role of maternal breastfeeding knowledge as a mediator in this relationship remains untested. Integrating self-efficacy theory with the KAP framework in a father–mother dyadic context extends beyond the predominantly mother-centered perspective of prior research. The Chinese postpartum context, characterized by the traditional zuoyuezi practice and intensive family involvement, provides a distinctive setting for examining this pathway.

In summary, grounded in Bandura’s self-efficacy theory and the KAP framework, this study targeted mothers and their partners at 6 weeks postpartum, with the following aims: (1) to examine the association between paternal breastfeeding support self-efficacy (PBSSE) and maternal breastfeeding behavior at 6 weeks postpartum; and (2) to determine whether maternal breastfeeding knowledge mediates this relationship. Based on the integrated theoretical framework described above, the following hypotheses were proposed: H1: PBSSE is positively associated with maternal breastfeeding behavior at 6 weeks postpartum. H2: PBSSE is positively associated with maternal breastfeeding knowledge. H3: Maternal breastfeeding knowledge mediates the association between PBSSE and breastfeeding behavior at 6 weeks postpartum.

## 2 Materials and methods

### 2.1 Study design and participants

A cross-sectional survey design was employed. A convenience sampling method was employed, recruiting consecutive eligible participants during the study period. Participants were mothers attending their six-week postpartum checkup and their partners, recruited between February and December 2024 from a tertiary Grade-A maternal and child specialty hospital in Fujian Province, China. Inclusion criteria were: (1) mothers attending the six-week postpartum examination and their partners; (2) aged 18 years or older; (3) mothers capable of normal breastfeeding; and (4) provision of informed consent to participate. Exclusion criteria were: (1) mothers or infants with serious medical conditions affecting breastfeeding; and (2) individuals with psychiatric disorders or cognitive impairment. This study was approved by the hospital ethics committee (Approval No.: 2024KY067) in accordance with the Declaration of Helsinki.

### 2.2 Sample size

The sample size was determined based on the requirements for structural equation modeling (SEM), following the recommendation by Kline (2015) that the ratio of observed variables to sample size should be no less than 10:1. The SEM in this study included 12 observed indicators (3 + 6 + 3 item parcels), yielding a minimum required sample of 120 cases. Accounting for a 20% invalid response rate, the target sample size was set at no fewer than 144 cases. A total of 480 questionnaires were distributed; 26 invalid questionnaires were excluded (due to uniform response patterns, excessively short completion times, or identification number anomalies), resulting in 454 valid responses and an effective response rate of 94.6%. Electronic questionnaires were used with all items set as mandatory; consequently, there were no missing data.

### 2.3 Instruments

#### 2.3.1 Demographic questionnaire

A researcher-designed questionnaire was used to collect general demographic information, including: age, ethnicity, educational level, occupation type, household income, maternity leave duration, parity, mode of delivery, history of gestational diabetes mellitus (GDM), labor analgesia, infant sex, nipple condition, postpartum confinement center experience, and prior breastfeeding education.

#### 2.3.2 Paternal Breastfeeding Support Self-Efficacy Scale

The Paternal Breastfeeding Support Self-Efficacy Scale was based on paternal breastfeeding self-efficacy measurement work among fathers (Chipojola et al., 2022; Dennis et al., 2018) and comprises 14 items rated on a 5-point Likert scale (1 = not at all confident to 5 = completely confident). Total scores range from 14 to 70, with higher scores indicating greater self-efficacy. The Cronbach’s α for this scale in the present study was 0.961.

#### 2.3.3 Maternal Breastfeeding Behavior Scale for the Puerperium

The Maternal Breastfeeding Behavior Scale for the Puerperium (Wu et al., 2023) consists of 36 items rated on a 5-point Likert scale (1 = never to 5 = always). Total scores range from 36 to 180, with higher scores reflecting better breastfeeding behavior. The Cronbach’s α in the present study was 0.978.

#### 2.3.4 Breastfeeding Knowledge Questionnaire

This questionnaire, adapted from La Leche League International (LLLI), contains 17 items with three response options (“yes,” “no,” and “do not know”). Correct answers are scored 1 point, while incorrect or uncertain responses are scored 0, yielding a total score of 0–17. The Cronbach’s α in the present study was 0.820. The questionnaire has been validated for cross-cultural use in diverse settings (Hailu et al., 2020).

### 2.4 Data analysis

Survey data were directly imported into Excel and independently checked and cleaned by two researchers. Statistical analyses were performed using SPSS 26.0. Categorical variables were described using frequencies and proportions. Continuous variables were assessed for normality using the Shapiro–Wilk test and skewness/kurtosis indices, and were expressed as mean ± standard deviation (M ± SD). Independent-samples *t*-tests or one-way analysis of variance (ANOVA) were used to compare breastfeeding behavior scores across demographic subgroups. Pearson correlation analysis was used to examine associations among variables, with Spearman rank correlation conducted as a sensitivity analysis to verify the robustness of results. Multiple linear regression was performed to identify factors associated with breastfeeding behavior, with variance inflation factors (VIF) calculated to assess multicollinearity. Harman’s single-factor test was used to evaluate common method bias.

Structural equation modeling was conducted using the lavaan package (version 0.6–19) in R 4.3.1. Item parceling was employed to construct indicators for the three latent variables: PBSSE (3 parcels: items 1–5, 6–10, and 11–14), maternal breastfeeding behavior at 6 weeks postpartum (6 parcels: 6 items per parcel), and breastfeeding knowledge (3 parcels: items 1–6, 7–12, and 13–17). Model parameters were estimated using maximum likelihood (ML), and mediation effects were tested using the bootstrap method (5,000 resamples) to calculate confidence intervals. Parity and whether family members had received breastfeeding health education were controlled as covariates in the structural model. The significance level was set at *α* = 0.05.

## 3 Results

### 3.1 General characteristics of participants

Among the 454 mothers at 6 weeks postpartum, 384 (84.6%) were aged <35 years and 70 (15.4%) were aged ≥35 years. The majority were of Han ethnicity (*n* = 438, 96.5%). Regarding educational attainment, 249 (54.8%) held a bachelor’s degree. A total of 312 (68.7%) were employed full-time. The most common household per capita monthly income bracket was 5,000–9,999 RMB (45.4%). With respect to maternity leave duration, 312 (68.7%) reported a leave period of 42 days to 6 months. Primiparous women accounted for 294 (64.8%) of the sample, and 276 (60.8%) delivered vaginally. A total of 343 (75.6%) had no history of gestational diabetes mellitus (GDM), and 358 (78.9%) received analgesic medication during delivery. Among the infants, 236 (52.0%) were male. Regarding nipple condition, 393 (86.6%) reported normal nipples bilaterally. The majority of participants (*n* = 384, 84.6%) had not stayed in a postpartum care center. A total of 255 (56.2%) had received breastfeeding-related health education, and 186 (41.0%) reported that their family members had received breastfeeding health education. Detailed demographic characteristics are presented in Table 1.

**Table 1:** Comparison of breastfeeding behavior scores at 6 weeks postpartum by maternal characteristics (*n* = 454).

Variable	Category	*n* (%)	Score (M ± SD)	Statistic	*p*	Effect size
Age	<35 years	384 (84.6)	145.42 ± 26.12	*t* = 1.223	0.222	*d* = 0.159
	≥35 years	70 (15.4)	149.66 ± 29.51			
Parity	Primiparous	294 (64.8)	143.55 ± 25.38	*t* = −2.746	0.006^**^	*d* = 0.270
	Multiparous	160 (35.2)	150.70 ± 28.41			
Nipple condition	Normal bilateral	393 (86.6)	147.40 ± 26.34	*F* = 4.147	0.016^*^	*η*^2^ = 0.018
	Unilateral inversion	38 (8.4)	140.05 ± 27.50			
	Bilateral inversion	23 (5.1)	133.30 ± 27.49			
Mode of delivery	Vaginal	276 (60.8)	147.85 ± 26.46	*t* = −1.770	0.077	*d* = 0.170
	Cesarean	178 (39.2)	143.32 ± 26.85			
BF education (mother)	Yes	255 (56.2)	148.67 ± 27.16	*t* = −2.364	0.019^*^	*d* = 0.224
	No	199 (43.8)	142.74 ± 25.73			
BF education (family)	Yes	186 (41.0)	150.99 ± 27.11	*t* = −3.307	0.001^**^	*d* = 0.316
	No	268 (59.0)	142.66 ± 25.88			

BF, breastfeeding. Only variables with *p* < 0.10 are shown. ^*^p < 0.05, ^**^p < 0.01.

### 3.2 Descriptive statistics

The mean scores for the three instruments were as follows: paternal breastfeeding support self-efficacy (PBSSE) 56.29 ± 10.79, maternal breastfeeding behavior at 6 weeks postpartum 146.04 ± 26.63, and breastfeeding knowledge 14.01 ± 2.87 (Table 2).

**Table 2:** Descriptive statistics of study instruments (*n* = 454).

Scale	Items	Range	Mean	SD	Median	Cronbach’s *α*
PBSSE	14	14–70	55.45	11.01	56.0	0.961
Breastfeeding behavior	36	36–180	146.07	26.68	146.0	0.978
Breastfeeding knowledge	17	0–17	14.00	3.04	15.0	0.820

PBSSE, Paternal Breastfeeding Support Self-Efficacy.

Normality testing using the Shapiro–Wilk test indicated that the total scores of all three scales were non-normally distributed (*p* < 0.001). Specifically, the PBSSE scale exhibited a skewness of −0.574 and kurtosis of 0.001; the maternal breastfeeding behavior scale showed a skewness of −1.222 and kurtosis of 2.094; and the breastfeeding knowledge scale demonstrated a skewness of −1.441 and kurtosis of 1.778. Following the criteria proposed by Curran et al. (1996), distributions with absolute skewness values <2 and absolute kurtosis values <7 may be considered approximately normal. Given the large sample size in the present study (*n* = 454), the central limit theorem further supports the use of parametric testing methods.

Regarding common method bias, Harman’s single-factor test revealed that the first unrotated factor accounted for 39.33% of the variance, which is below the 40% critical threshold, indicating that common method bias was not a serious concern in this study. With respect to scale reliability, Cronbach’s α coefficients were 0.961 for the PBSSE scale, 0.978 for the maternal breastfeeding behavior scale, and 0.820 for the Breastfeeding Knowledge Questionnaire, all demonstrating good internal consistency.

### 3.3 Correlation analysis

Correlation analysis revealed that PBSSE was significantly and positively correlated with maternal breastfeeding behavior at 6 weeks postpartum (Pearson *r* = 0.523, Spearman *ρ* = 0.351, *p* < 0.01) and with breastfeeding knowledge (Pearson *r* = 0.500, Spearman *ρ* = 0.392, *p* < 0.01). Breastfeeding knowledge was also significantly and positively correlated with breastfeeding behavior at 6 weeks postpartum (Pearson *r* = 0.445, Spearman *ρ* = 0.299, *p* < 0.01). The Spearman rank correlation coefficients were slightly lower than the Pearson coefficients, which may be attributable to a ceiling effect in the breastfeeding knowledge scale; however, the positive associations among all variables remained consistent and statistically significant across both methods (Table 3).

**Table 3:** Correlation analysis among study variables (*n* = 454).

Variable	1	2	3
Pearson *r*			
1. PBSSE	–		
2. Breastfeeding knowledge	0.500^**^	–	
3. Breastfeeding behavior	0.523^**^	0.445^**^	–
Spearman *ρ*			
1. PBSSE	–		
2. Breastfeeding knowledge	0.392^**^	–	
3. Breastfeeding behavior	0.351^**^	0.299^**^	–

^**^p < 0.01. Upper panel: Pearson *r*; lower panel: Spearman *ρ* (sensitivity analysis). PBSSE, Paternal Breastfeeding Support Self-Efficacy.

### 3.4 Multiple regression analysis

Multiple linear regression was conducted with maternal breastfeeding behavior at 6 weeks postpartum as the dependent variable. Independent variables included demographic factors that reached statistical significance in univariate analyses (nipple condition, parity, receipt of breastfeeding health education, and family members’ receipt of breastfeeding health education), as well as PBSSE and breastfeeding knowledge. The results indicated that bilateral inverted nipples (*β* = −0.089, *p* = 0.020), primiparity (*β* = −0.084, *p* = 0.029), family members having received breastfeeding health education (*β* = 0.141, *p* = 0.007), PBSSE (*β* = 0.396, *p* < 0.001), and breastfeeding knowledge (*β* = 0.234, *p* < 0.001) were significant predictors of maternal breastfeeding behavior at 6 weeks postpartum. Collectively, these variables explained 34.5% of the variance in breastfeeding behavior (adjusted *R*^2^ = 0.345, *F* = 35.086, *p* < 0.001). All variance inflation factor (VIF) values were <2.0, confirming the absence of multicollinearity (Table 4).

**Table 4:** Multiple linear regression analysis of factors associated with breastfeeding behavior (*n* = 454).

Predictor	*B*	SE	*β*	*t*	*p*	VIF
(Constant)	46.792	8.231	–	5.687	<0.001	–
Bilateral nipple inversion	−12.526	5.386	−0.089	−2.325	0.020	1.080
Primiparity	−4.618	2.114	−0.084	−2.185	0.029	1.091
Family BF education (yes)	7.802	2.888	0.141	2.702	0.007	1.151
PBSSE	0.960	0.105	0.396	9.171	<0.001	1.391
Breastfeeding knowledge	2.050	0.387	0.234	5.295	<0.001	1.460

*R*^2^ = 0.355, adjusted *R*^2^ = 0.345, *F* = 35.086, *p* < 0.001. BF, breastfeeding; PBSSE, Paternal Breastfeeding Support Self-Efficacy; VIF, variance inflation factor.

### 3.5 Mediation analysis

A structural equation model (SEM) was constructed using the lavaan package in R 4.3.1 to examine the mediating role of breastfeeding knowledge in the relationship between PBSSE and maternal breastfeeding behavior at 6 weeks postpartum. Item parceling was employed to create indicators for the three latent variables: three parcels for PBSSE (items 1–5, 6–10, and 11–14), six parcels for maternal breastfeeding behavior (six items per parcel), and three parcels for breastfeeding knowledge (items 1–6, 7–12, and 13–17). Parity and family members’ receipt of breastfeeding health education were included as covariates in the structural model.

Measurement model validation showed that all factor loadings were significant (*p* < 0.001), with standardized loadings ranging from 0.493 to 0.943 (Table 5). The overall model demonstrated good fit: *χ*^2^/*df* = 3.547, GFI = 0.914, CFI = 0.964, TLI = 0.954, RMSEA = 0.075 (90% CI: 0.065–0.085), SRMR = 0.052 (Table 6).

**Table 5:** Standardized factor loadings of the measurement model.

Latent variable	Indicator	Standardized loading	95% bootstrap CI
PBSSE	Parcel 1 (items 1–5)	0.897	(reference)
	Parcel 2 (items 6–10)	0.943	[0.948, 1.081]
	Parcel 3 (items 11–14)	0.874	[0.933, 1.076]
Breastfeeding behavior	Parcel 1 (items 1–6)	0.862	(reference)
	Parcel 2 (items 7–12)	0.940	[1.010, 1.116]
	Parcel 3 (items 13–18)	0.942	[0.984, 1.112]
	Parcel 4 (items 19–24)	0.923	[0.943, 1.076]
	Parcel 5 (items 25–30)	0.867	[0.898, 1.028]
	Parcel 6 (items 31–36)	0.810	[0.893, 1.049]
Breastfeeding knowledge	Parcel 1 (items 1–6)	0.727	(reference)
	Parcel 2 (items 7–12)	0.854	[1.016, 1.381]
	Parcel 3 (items 13–17)	0.493	[0.385, 0.729]

All factor loadings *p* < 0.001. The first indicator of each latent variable was fixed to 1.0 (reference). CI, confidence interval for the unstandardized loading; PBSSE, Paternal Breastfeeding Support Self-Efficacy.

**Table 6:** Goodness-of-fit indices for the structural equation model.

Fit index	Value	Criterion	Evaluation
*χ*^2^/*df*	3.547	<5 acceptable	Acceptable
GFI	0.914	>0.90 acceptable	Acceptable
CFI	0.964	>0.95 good	Good
TLI	0.954	>0.95 good	Good
RMSEA	0.075	<0.08 good	Good
RMSEA 90% CI	0.065–0.085		
SRMR	0.052	<0.08 good	Good

GFI, goodness-of-fit index; CFI, comparative fit index; TLI, Tucker–Lewis index; RMSEA, root mean square error of approximation; SRMR, standardized root mean square residual.

Path analysis of the structural model (Figure 1) revealed that the direct effect of PBSSE on maternal breastfeeding behavior at 6 weeks postpartum was significant (*β* = 0.412, 95% CI: 0.236–0.527, *p* < 0.001). The path from PBSSE to breastfeeding knowledge was also significant (*β* = 0.593, *p* < 0.001), as was the path from breastfeeding knowledge to breastfeeding behavior (*β* = 0.221, *p* = 0.015). Additionally, parity (*β* = 0.088, *p* = 0.029) and family members’ receipt of breastfeeding health education (*β* = 0.142, *p* < 0.001) exerted significant direct effects on breastfeeding behavior.

**Figure 1:**
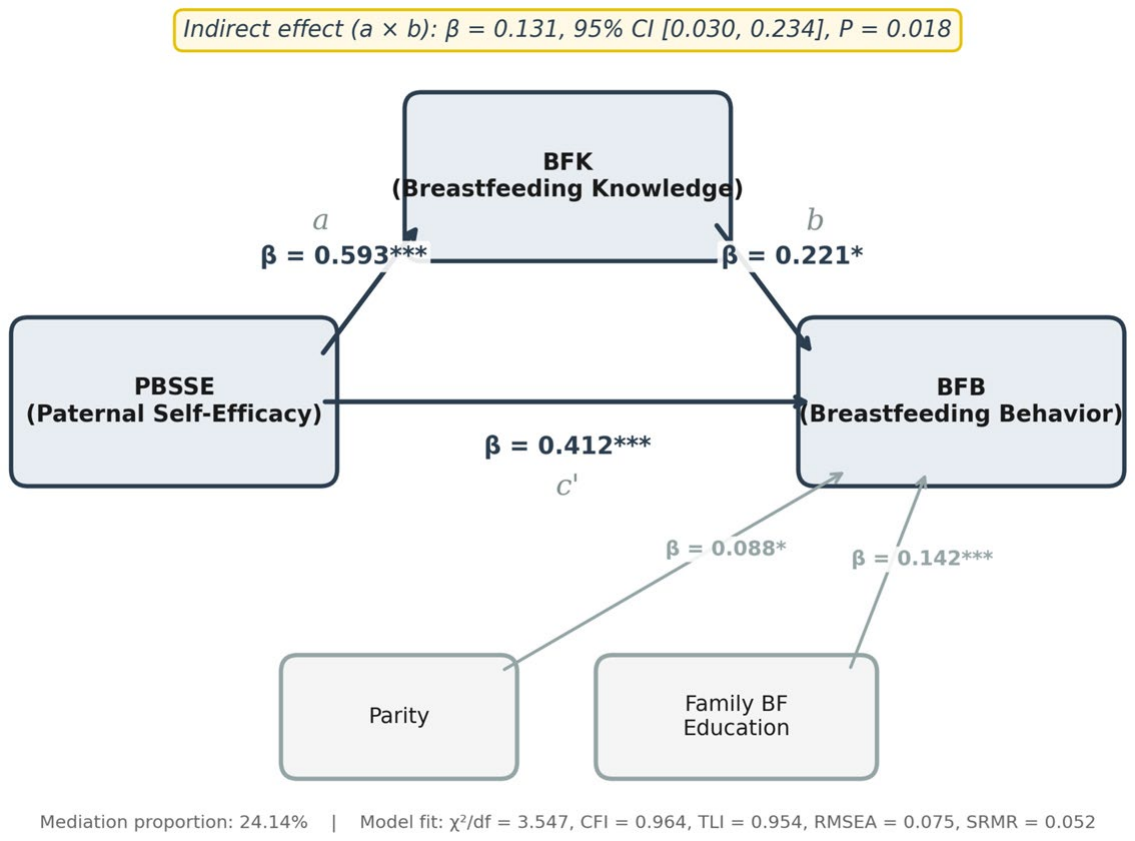
Structural equation model of the mediating effect of breastfeeding knowledge in the relationship between paternal breastfeeding support self-efficacy (PBSSE) and maternal breastfeeding behavior at 6 weeks postpartum. Standardized path coefficients are shown. Parity and family breastfeeding health education were included as covariates. *p < 0.05, **p < 0.01, ***p < 0.001.

Bootstrap mediation testing (Table 7) demonstrated a significant indirect effect of PBSSE on maternal breastfeeding behavior at 6 weeks postpartum through breastfeeding knowledge (*β* = 0.131, 95% CI: 0.030–0.234, *p* = 0.018), with the mediating effect accounting for 24.14% of the total effect. As the direct effect was also significant (*β* = 0.412, *p* < 0.001), breastfeeding knowledge served as a partial mediator in the relationship between PBSSE and maternal breastfeeding behavior at 6 weeks postpartum.

**Table 7:** Bootstrap test of mediation effects (*n* = 454, 5,000 resamples).

Effect path	Standardized effect	SE	95% bootstrap CI	*p*
Total effect (*c*)	0.543	0.058	[0.396, 0.620]	<0.001
Direct effect (*c*′)	0.412	0.074	[0.236, 0.527]	<0.001
Indirect effect (*a* × *b*)	0.131	0.052	[0.030, 0.234]	0.018
Mediation proportion	24.14%	–	[5.8, 47.5%]	0.022

Path *a* = PBSSE → breastfeeding knowledge (*β* = 0.593); path *b* = breastfeeding knowledge → breastfeeding behavior (*β* = 0.221). Covariates controlled: parity, family breastfeeding education. PBSSE, Paternal Breastfeeding Support Self-Efficacy.

## 4 Discussion

This study examined the association between paternal breastfeeding support self-efficacy (PBSSE) and maternal breastfeeding behavior at 6 weeks postpartum, and further analyzed the mediating role of maternal breastfeeding knowledge, building upon existing literature. Our findings indicate a significant positive correlation between PBSSE and maternal breastfeeding behavior at 6 weeks postpartum. Furthermore, maternal breastfeeding knowledge partially mediated this relationship, representing an indirect associational pathway between PBSSE and breastfeeding behavior through maternal breastfeeding knowledge.

Building upon existing research that has primarily focused on maternal factors, this study advances the field in two respects: first, by positioning PBSSE as a core predictor variable rather than a peripheral support factor; and second, by testing maternal breastfeeding knowledge as a mediating mechanism linking paternal self-efficacy to maternal behavior—a theoretically derived pathway that has not been directly examined in the existing literature. The following sections discuss each main finding in the context of prior evidence and the specific sociocultural setting of this study.

Our results demonstrated that PBSSE was significantly and positively correlated with maternal breastfeeding behavior at 6 weeks postpartum (*p* < 0.01); that is, higher levels of paternal self-efficacy were associated with more favorable maternal breastfeeding behaviors. This finding is consistent with the work of Brown and Davies (2014) and is further supported by a recent narrative review confirming that paternal psychosocial support is positively associated with breastfeeding duration (Fan et al., 2025). According to Bandura’s self-efficacy theory, fathers with high self-efficacy are more inclined to translate their internal beliefs into concrete supportive behaviors, such as actively assisting with breastfeeding and assuming childcare responsibilities, which may create favorable conditions for breastfeeding (Bandura, 1977; Susin and Giugliani, 2008). Additionally, active paternal support is associated with greater maternal breastfeeding confidence, alleviation of breastfeeding-related anxiety, and enhanced intrinsic motivation to continue breastfeeding (Tohotoa et al., 2011). Notably, the first 6 weeks postpartum represent a critical transitional period from breastfeeding initiation to maintenance. Effective paternal support during this period not only provides practical assistance but also conveys emotional validation and shared responsibility, which may help mothers overcome common challenges such as nipple pain and perceived insufficient milk supply (Dennis and Faux, 1999; Odom et al., 2013). Conversely, fathers with low self-efficacy may suggest supplementation with formula, potentially undermining maternal commitment to exclusive breastfeeding (Mitchell-Box and Braun, 2012). In the Chinese sociocultural context, the “zuoyuezi” (sitting the month) tradition means that mothers typically remain at home for the first four to 6 weeks postpartum, during which the father’s daily involvement and confidence may be particularly influential on feeding decisions. Unlike Western settings where mothers may resume social activities sooner, this concentrated home-based recovery period may amplify the father’s proximal support role. Recent evidence indicates that exclusive breastfeeding rates at 6 weeks postpartum remain low in Chinese settings (35.28%; Wang et al., 2024), underscoring the need for effective support mechanisms during this period. However, maternal breastfeeding self-efficacy (BFSE) was not measured in this study, despite being among the strongest known predictors of breastfeeding behavior. The observed PBSSE–BFB association may partially reflect shared family-level factors, as couples with strong breastfeeding commitment may simultaneously exhibit high PBSSE and high maternal BFSE, a pattern consistent with Sezer et al. (2025), who reported a significant positive correlation between paternal and maternal breastfeeding self-efficacy (*r* = 0.520); future studies should include maternal BFSE to disentangle these effects. Taken together, PBSSE represents an important protective factor positively associated with breastfeeding behavior in the early postpartum period.

This study also found that PBSSE was significantly and positively correlated with maternal breastfeeding knowledge (*p* < 0.01); specifically, higher paternal self-efficacy was associated with greater maternal breastfeeding knowledge. This result is consistent with the findings of Zeng et al. (2024), who similarly confirmed a positive association between paternal breastfeeding knowledge and self-efficacy in a multicenter cross-sectional survey conducted in southeastern China. Regarding the underlying mechanism, Bandura’s self-efficacy theory (Bandura, 1977) posits that individuals with high self-efficacy are more inclined to actively seek information related to specific behaviors. In the breastfeeding context, when fathers possess greater confidence in their ability to support breastfeeding, they are more likely to proactively acquire breastfeeding-related knowledge—such as lactation techniques and management of common problems—and share this information with their partners through daily communication, which may be associated with higher maternal knowledge levels (Chipojola et al., 2025). Furthermore, Chipojola et al. (2025) found significant positive correlations between maternal and paternal breastfeeding self-efficacy in a dyadic study in Malawi, suggesting that paternal self-efficacy may positively relate to maternal cognitive resources through enhanced supportive interactions and information exchange between partners. This mechanism has also received preliminary support in studies of fathers of preterm infants (Denoual et al., 2016). In contemporary China, social media platforms such as WeChat parenting groups and short-video applications (e.g., Xiaohongshu, Douyin) have become major channels for acquiring parenting knowledge. Fathers with higher self-efficacy may be more likely to engage with these digital resources and share relevant content with their partners, representing a digitally mediated knowledge pathway that has received limited attention in Western research. However, the positive PBSSE–BFK correlation may also reflect confounding by education level and socioeconomic status, as more educated families may simultaneously exhibit higher paternal self-efficacy and greater maternal knowledge; future research should control for these potential confounders.

Another important finding of this study is the identification of an indirect associational pathway linking PBSSE to maternal breastfeeding knowledge and subsequently to breastfeeding behavior at 6 weeks postpartum. Specifically, fathers with higher self-efficacy tend to proactively acquire evidence-based breastfeeding knowledge—including lactation physiology, correct latch techniques, and strategies for managing common difficulties—and transmit this knowledge to mothers through daily communication and interaction (Chipojola et al., 2025; Susin and Giugliani, 2008). This knowledge transfer process is associated with enhanced maternal breastfeeding knowledge, which may be related to mothers’ understanding of feeding patterns at a cognitive level and acquisition of practical skills, potentially contributing to more informed responses to breastfeeding challenges and higher levels of feeding confidence and behavioral persistence (Abbass-Dick et al., 2015). This is particularly relevant during the first 6 weeks postpartum, a critical adaptation phase during which mothers are establishing and consolidating breastfeeding behaviors; knowledge support is associated with mothers more rapidly establishing stable, evidence-based feeding patterns (Mitchell-Box and Braun, 2012; Odom et al., 2013). Previous research, including a large Chinese cross-sectional study (*n* = 3,738), has confirmed that maternal breastfeeding knowledge is a significant protective factor for exclusive breastfeeding (Chen et al., 2023). Therefore, breastfeeding knowledge serves as a cognitive mediator in this pathway. This indirect pathway suggests that paternal support is associated with breastfeeding behavior not only through emotional encouragement and practical assistance but also through an indirect knowledge-empowerment mechanism, reflecting the link between “willingness to support” and “effective support.” Notably, the mediation effect accounted for only 24.14% of the total effect, indicating that approximately 76% of PBSSE’s association with breastfeeding behavior operates through pathways other than maternal knowledge—potentially including emotional support (anxiety reduction), practical assistance (sharing childcare and household tasks), attitudinal alignment (shared commitment to breastfeeding), and help-seeking facilitation (accompanying mothers to postpartum consultations). Furthermore, the relatively wide 95% CI (0.030–0.234) for the indirect effect suggests considerable variability in the mediation pathway, possibly attributable to the ceiling effect of the BFK scale (mean 14.0/17), which restricts variability in the mediator and may attenuate mediation estimates. It should also be acknowledged that the cross-sectional design precludes establishing temporal precedence. A plausible reverse pathway exists: mothers who breastfeed successfully may accumulate knowledge through experience, which may in turn reinforce their partners’ confidence. Future longitudinal studies measuring these variables at prenatal, one-week, and six-week postpartum time points are needed to establish directionality—an approach exemplified by Liu et al. (2025), who used cross-lagged panel models to demonstrate that breastfeeding self-efficacy mediates the relationship between perinatal depressive symptoms and exclusive breastfeeding over time.

It is noteworthy that parity was significantly associated with breastfeeding behavior at 6 weeks postpartum (*p* = 0.006), with multiparous women scoring higher than primiparous women on breastfeeding behavior (Cohen’s *d* = 0.270). This finding is consistent with prior research and may be attributable to the breastfeeding experience of multiparous women; previous successful breastfeeding experiences are associated with enhanced maternal feeding confidence and skill mastery, suggesting that primiparous women require additional breastfeeding support and guidance. Clinically, primiparous women and their partners should be prioritized for antenatal breastfeeding preparedness programs, and peer support mechanisms pairing experienced mothers with first-time mothers could be explored. Moreover, as China’s three-child policy increases the proportion of multiparous women, targeted support for primiparous mothers remains essential, as their initial breastfeeding experience shapes attitudes toward feeding in subsequent pregnancies.

Additionally, family members having received breastfeeding health education was significantly and positively associated with maternal breastfeeding behavior at 6 weeks postpartum (*p* = 0.001), and remained an independent predictor in the multiple regression model (*β* = 0.141, *p* = 0.007) as well as in the structural equation model (*β* = 0.142, *p* < 0.001). This finding further supports the importance of whole-family participation in breastfeeding education. Not only is paternal self-efficacy associated with breastfeeding behavior, but the knowledge base of family members is similarly related, suggesting that clinical interventions should expand from a mother-centered to a family-centered breastfeeding health education model. In China, grandparents—particularly mothers-in-law—typically provide intensive daily care during the postpartum confinement period, and their feeding beliefs directly influence maternal practices; for instance, uninformed family members may encourage early formula supplementation or premature introduction of complementary foods. Whether family members have received evidence-based breastfeeding education therefore shapes whether the home environment facilitates or undermines breastfeeding. We suggest that antenatal breastfeeding education programs should include primary family caregivers (partners and grandparents) rather than targeting mothers alone, consistent with the WHO-recommended family-centered care model, and supported by recent experimental evidence showing that father-targeted breastfeeding education significantly increased optimal breastfeeding practices (RR = 1.38; Abageda et al., 2025). Furthermore, couple-based interventions such as personalized text-message programs have demonstrated positive effects on breastfeeding self-efficacy (Henshaw et al., 2024).

However, this study has several limitations. First, due to the cross-sectional design, causal relationships between variables cannot be established. The mediation analysis results should be interpreted as statistical associational pathways rather than causal mechanisms, and the possibility of reverse causation cannot be excluded. These findings require further verification through future longitudinal or interventional studies. Second, data on paternal self-efficacy, maternal knowledge, and breastfeeding behavior were all obtained through participant self-report, which may be subject to recall bias or social desirability bias, potentially affecting the precision of certain measurements. Future research should incorporate objective assessment methods where possible. Third, the breastfeeding knowledge scale exhibited a ceiling effect (58.6% of participants scored ≥15/17), which may have restricted the range of variability and consequently affected the precision of mediation effect estimates. Fourth, maternal breastfeeding self-efficacy was not included as a covariate in this study, despite being established as one of the strongest predictors of breastfeeding behavior; future research should incorporate this variable to construct a more comprehensive theoretical model. Fifth, paternal breastfeeding knowledge was not measured, precluding differentiation between a direct knowledge transfer effect of paternal self-efficacy on maternal knowledge and other indirect mechanisms. Sixth, the study sample was drawn from a single hospital using convenience sampling, limiting generalizability. Future research should employ multicenter stratified sampling strategies with expanded sample sizes to enhance external validity.

## 5 Conclusion

This study found that paternal breastfeeding support self-efficacy was positively associated with maternal breastfeeding behavior at 6 weeks postpartum, with maternal breastfeeding knowledge partially mediating this relationship. Additionally, parity and family members having received breastfeeding education were identified as factors associated with breastfeeding behavior at 6 weeks postpartum. These findings suggest that clinical practice focusing on and supporting paternal breastfeeding self-efficacy, while concurrently strengthening breastfeeding knowledge education for mothers and family members, may contribute to optimizing breastfeeding behavior in the early postpartum period. Future longitudinal research is needed to further verify this associational pathway.

## Data Availability

The raw data supporting the conclusions of this article will be made available by the authors, without undue reservation.
